# Novel receptor targets for production and action of allopregnanolone in the central nervous system: a focus on pregnane xenobiotic receptor

**DOI:** 10.3389/fncel.2014.00106

**Published:** 2014-04-09

**Authors:** Cheryl A. Frye, Carolyn J. Koonce, Alicia A. Walf

**Affiliations:** ^1^Department of Psychology, The University at Albany-SUNYAlbany, NY, USA; ^2^Department of Biological Sciences, The University at Albany-SUNYAlbany, NY, USA; ^3^The Centers for Neuroscience, The University at Albany-SUNYAlbany, NY, USA; ^4^Life Sciences Research, The University at Albany-SUNYAlbany, NY, USA; ^5^Department of Chemistry and Biochemistry, The University of Alaska–FairbanksFairbanks, AK, USA; ^6^Institute of Arctic Biology, The University of Alaska–FairbanksFairbanks, AK, USA; ^7^IDeA Network of Biomedical Excellence (INBRE), The University of Alaska–FairbanksFairbanks, AK, USA

**Keywords:** midbrain, ventral tegmental area, allopregnanolone, neurosteroid, reproduction, pregnane xenobiotic receptor, non-genomic

## Abstract

Neurosteroids are cholesterol-based hormones that can be produced in the brain, independent of secretion from peripheral endocrine glands, such as the gonads and adrenals. A focus in our laboratory for over 25 years has been how production of the pregnane neurosteroid, allopregnanolone, is regulated and the novel (i.e., non steroid receptor) targets for steroid action for behavior. One endpoint of interest has been lordosis, the mating posture of female rodents. Allopregnanolone is necessary and sufficient for lordosis, and the brain circuitry underlying it, such as actions in the midbrain ventral tegmental area (VTA), has been well-characterized. Published and recent findings supporting a dynamic role of allopregnanolone are included in this review. First, contributions of ovarian and adrenal sources of precursors of allopregnanolone, and the requisite enzymatic actions for *de novo* production in the central nervous system will be discussed. Second, how allopregnanolone produced in the brain has actions on behavioral processes that are independent of binding to steroid receptors, but instead involve rapid modulatory actions via neurotransmitter targets (e.g., γ-amino butyric acid-GABA, N-methyl-D-aspartate- NMDA) will be reviewed. Third, a recent focus on characterizing the role of a promiscuous nuclear receptor, pregnane xenobiotic receptor (PXR), involved in cholesterol metabolism and expressed in the VTA, as a target for allopregnanolone and how this relates to both actions and production of allopregnanolone will be addressed. For example, allopregnanolone can bind PXR and knocking down expression of PXR in the midbrain VTA attenuates actions of allopregnanolone via NMDA and/or GABA_A_ for lordosis. Our understanding of allopregnanolone’s actions in the VTA for lordosis has been extended to reveal the role of allopregnanolone for broader, clinically-relevant questions, such as neurodevelopmental processes, neuropsychiatric disorders, epilepsy, and aging.

## Introduction

Now it is generally understood that cholesterol-based hormones (“steroids”) can be produced in the brain and peripheral nerves and not only in traditional steroid organs, such as the ovaries, adrenals, and placenta. This notion is based on initial findings by Baulieu and colleagues in the early eighties, and further supported by decades of follow-up studies (Baulieu, 1980, 1991). These steroids, which are produced in the brain and the peripheral nerves, were given the name “neurosteroids” to differentiate them from the same steroids that are produced by peripheral glands. These initial discoveries demonstrated that precursors to the pregnane steroids, such as pregnenolone were greater in the brain and peripheral nerves, than in circulation. As well, the same steroidogenic enzymes in the peripheral steroid gland were found to be expressed in the nervous system and involved in production of these molecules (Compagnone and Mellon, 2000; Furukawa et al., 2002). These steroids measured in the brain may be also products of metabolism of peripheral organ derived precursors; these molecules are referred to as “neuroactive” steroids. The pregnane steroid, 5α-pregnan-3α-ol-20-one (a.k.a. allopregnanolone or 3α,5α-THP) will be the focus herein. Levels of allopregnanolone in the nervous system can be much greater than circulating levels, and even persist after removal of the glands that produce pregnane steroids in the body (i.e., following ovariectomy—OVX and/or adrenalectomy—ADX). Indeed, these and other studies substantiated the notion that allopregnanolone is synthesized *de novo* in the brain and peripheral nerves, and that levels in the nervous system are not only a product of metabolism from peripheral gland-derived precursors and subsequent accumulation in neural tissues (Baulieu, 1980, 1991; Majewska, 1992; Paul and Purdy, 1992; Mellon, 1994). A central question in our laboratory has been in determining the extent to which allopregnanolone’s functional effects are related to its synthesis in the brain, and/or metabolism of its precursors from the periphery (e.g., progesterone), in the brain. A brief summary of the key information supporting the role of allopregnanolone as a neurosteroid and neuroactive steroid is as follows.

There are highly coordinated actions of steroidogenic enzymes in neurons and glia in regions of the brain supporting production of allopregnanolone as a neuroactive steroid and neurosteroid. The brain and peripheral nerves express all of the enzymes required for metabolism or biosynthesis of allopregnanolone (Compagnone and Mellon, 2000). Regarding metabolism, circulating progesterone, secreted from peripheral glands, can be sequestered and accumulated in the brain, and then can be metabolized by enzymes to other neuroactive metabolites. Formation of allopregnanolone from progesterone is dependent upon sequential actions of 5α-reductase (which produces dihydroprogesterone), and then 3α-hydroxysteroid dehydrogenase (3α-HSD). Additionally, allopregnanolone can be formed from biosynthesis in the brain itself (Baulieu, 1991; Paul and Purdy, 1992; Mellon, 1994; King et al., 2002; Papadopoulos et al., 2006a,b; Batarseh and Papadopoulos, 2010). The requisite factors for allopregnanolone biosynthesis involves the 18kDA translocator protein (TSPO, formerly known as the mitochondrial benzodiazepine receptor or the peripheral-type benzodiazepine receptor), which binds cholesterol at high affinity. TSPO, with the steroidogenic acute regulatory (StAR) protein, have actions to transport cholesterol into mitochondria, which is considered a rate-limiting step for allopregnanolone biosynthesis (Mellon and Deschepper, 1993; King et al., 2004; Papadopoulos et al., 2006a,b). Cholesterol is then oxidized to pregnenolone by cytochrome P450-dependent C27 side chain cleavage enzymes (P450scc), which is converted to progesterone by 3β-hydroxysteroid dehydrogenase enzymes. Progesterone from this biosynthesis, can then be converted to allopregnanolone by actions of 5α-reductase and 3α-HSD. As such, production of allopregnanolone can be from metabolism of circulating progesterone, or *de novo* production of progesterone in the nervous system. All of these factors involved in metabolism to, or biosynthesis of, allopregnanolone, described above, are expressed in the spinal cord, cerebellum, hindbrain (e.g., pons, medulla), midbrain (e.g., tegmentum), and forebrain (e.g., corticolimbic regions, such as prefrontal cortex and hippocampus, as well as basal ganglia, hypothalamus, and thalamus); however, there are differences in expression based upon many factors, including age, sex, hormonal milieu, cell type, context (Mellon, 2007; Frye, 2009). Nevertheless, the vast distribution of these factors, and their conservation across species (see review Mellon, 2007), implies the importance of neuro(active) steroids, such as allopregnanolone, for brain function, and supports investigations to understand the functional significance of allopregnanolone from metabolism and/or biosynthesis (Melcangi et al., 2014).

A focus in our laboratory for over 25 years has been how production of allopregnanolone is regulated, and the novel targets for allopregnanolone’s functional effects, including behavioral endpoints. This review will summarize early studies about challenge/stressor-induced biosynthesis of allopregnanolone and what is known about allopregnanolone synthesis and its actions from studies using mating as a manipulation and measure in our laboratory. Additionally, there will be a focus on recent studies, and inclusion of data in support, regarding the role of the pregnane xenobiotic receptor (PXR) as a novel factor for allopregnanolone synthesis and actions. Lastly, there will be a discussion of how these basic studies centered on allopregnanolone synthesis and action in the midbrain of rodents have been extended to clinically-relevant findings.

## Challenge-induced allopregnanolone synthesis

Early studies investigating allopregnanolone as a neurosteroid identified that environmental challenge, or stressors, can induce allopregnanolone biosynthesis. In support, acute cold-water swimming, an experimental model of an acute physical stressor in rodents, increases brain production of allopregnanolone (Purdy et al., 1991; Barbaccia et al., 1996; Vallée et al., 2000). Similar effects are observed with other acute stressors, such as footshock, ether exposure, and/or carbon dioxide exposure have been demonstrated in intact, gonadectomized/ovariectomized (OVX), and/or adrenalectomized (ADX) rodents (Paul and Purdy, 1992; Barbaccia et al., 1996). Analogous effects in intact rodents and those with peripheral sources of progesterone removed support allopregnanolone biosynthesis as a response to these challenges. Alternatively, allopregnanolone levels can be reduced following exposure to chronic laboratory stressors in adult rodents, such as social isolation (Serra et al., 2004; Agís-Balboa et al., 2007; Pibiri et al., 2008; Nin et al., 2011; Pinna and Rasmusson, 2012). Exposure to stressors of rodents *in utero* (e.g., immune challenges, restraint stress, immune challenges, exposure to cold, swim stress during the last week of gestation), or in early development (e.g., maternal deprivation) produces long-lasting reductions in allopregnanolone (Kellogg and Frye, 1999; Kehoe et al., 2000; McCormick et al., 2002; Paris and Frye, 2011; Paris et al., 2011a,b). There are functional effects of reducing allopregnanolone synthesis related to these responses in that greater stress responding is associated with lower levels of allopregnanolone (Zimmerberg and Blaskey, 1998; Frye and Walf, 2004; Agís-Balboa et al., 2007; Brunton and Russell, 2011; Paris et al., 2011a,b). Thus, it has been recognized for some time that extreme situations and behavioral experiences can alter allopregnanolone; however, there is now a greater understanding of robust effects of ecologically-relevant behavior, such as mating, on allopregnanolone synthesis.

## Mating as a manipulation and measure for investigating allopregnanolone synthesis and action in the brain

To facilitate further understanding of allopregnanolone’s functions and targets, and the role of its metabolism or biosynthesis, it has proved useful to focus on a behavior that is reliant upon allopregnanolone synthesis and actions (and subsequently extend this approach to other functions, described later in this review). In our laboratory, mating behavior of female rodents is thus utilized as both a manipulation and measure to elucidate allopregnanolone’s role. From studies using this approach, the importance of synthesis and actions of allopregnanolone in the midbrain ventral tegmental area (VTA) have been consistently revealed, and will be discussed in the following paragraphs.

### Mating behavior assessment

The midbrain VTA is known for its actions for motivated responses, and mating can be considered such a motivated behavior. Mating responses of females are quantified with measures of lordosis, proceptivity, and aggression. Lordosis, the necessary posture of female rodents for mating, can be quantified in the laboratory as the number of such responses by the female as a ratio (or quotient; lordosis quotients) of the attempts by the male. Other behaviors, such as proceptivity (courtship behaviors; proceptivity quotients) or aggression (rejection of males’ advances; aggression quotients) can be concurrently assessed with lordosis. As well, in our laboratory, we typically assess other behaviors beyond those directly related to mating, but those that may have consequences for successful reproduction, such as exploration, reductions in fear/anxiety, and social behavior with conspecifics (for review see Frye, 2009). Mating is a motivated behavior that is only observed under appropriate endocrine and environmental contexts, and one in which the brain circuitry necessary for it to occur (namely in the hypothalamus and midbrain for female rodents), and may modify its expression (e.g., corticolimbic structures), are becoming better characterized (DeBold and Malsbury, 1989; Frye and Walf, 2008; Pfaff et al., 2008; Frye, 2011).

### Allopregnanolone in the midbrain VTA is necessary and sufficient for mating

By utilizing this behavioral response of mating as a bioassay, we have been able to determine that allopregnanolone, from both metabolism of circulating progesterone, and biosynthesis in the midbrain, in the midbrain VTA is necessary and sufficient for mating (reviewed recently in Frye, 2011). Requisite enzymes and proteins for metabolism and biosynthesis of allopregnanolone are expressed in the midbrain VTA as well as in corticolimbic regions that may be involved (Cheng and Karavolas, 1975; Li et al., 1997; Furukawa et al., 2002; Frye, 2011; Frye et al., 2013a). Observations of age-related changes in reproductive behaviors and timing of reproductive senescence among female rats suggest that reductions in capacity to form allopregnanolone in the midbrain may be involved (Walf et al., 2011). Genetic knockout of 5α-reductase in female mice lowers allopregnanolone levels in the midbrain and attenuates lordosis during proestrous (when females typically have their highest levels of allopregnanolone, coincident with mating), and following ovariectomy and progesterone administration (Koonce and Frye, 2014). 5α-reductase knockout mice have normative responses to allopregnanolone administration. In addition to these findings that suggest the importance of progesterone metabolism in the midbrain for mating, there are data in support of the role of allopregnanolone synthesis in the VTA for mating. Antagonists of TSPO, P450scc, and 3β-HSD, delivered directly to the midbrain VTA, of receptive rats attenuates lordosis similarly as inhibitors of metabolism by 5α-reductase and 3α-HSD (reviewed in Frye, 2011; Frye et al., 2013a). As well, agonists of TSPO can have similar actions as allopregnanolone to OVX and ADX rats to increase midbrain levels of allopregnanolone and lordosis (reviewed in Frye, 2011; Frye et al., 2013a). Together, these approaches have suggested the importance of allopregnanolone, from both metabolism and biosynthesis, in the midbrain VTA for mating.

### Mating-induced allopregnanolone synthesis in the midbrain

In addition to being a measure of interest, mating can induce allopregnanolone formation in the nervous system, and, thereby, can be considered a manipulation as well. Among proestrous female rats that engage in mating with a male, there is a rapid increase in allopregnanolone levels in the midbrain; this same pattern of allopregnanolone synthesis is not observed with the smell, or site, of a sexually-experience male, or a female conspecific (Frye and Bayon, 1999; Frye et al., 2007). Notably, allopregnanolone levels are higher following “paced mating” compared to a standard mating task (Frye, 2001a,b, 2009, 2011; Frye et al., 2007, 2014b). Paced mating is considered a semi-naturalistic mating paradigm as compared to a standard mating paradigm, which is typically performed in a small chamber in a laboratory (e.g., a 10 gallon aquarium). Paced mating is considered closer to the natural experience because the chamber is larger and divided with an entry only a female can transverse to get to the other side of the chamber; as such, female rats can control the timing of (i.e., “pace”) their mating contacts with males, which is a critical part of the natural response in the wild and to enhance fertility and fecundity (Frye and Erskine, 1990). Even in the situation that females are tested in a large, paced mating chamber in the laboratory, but do not spontaneously pace, or show a low pacing response (but the same number of mounts by the male), there are lower levels of allopregnanolone in the midbrain compared to females that do show the pacing response (Frye and Rhodes, 2006). These data support the notion that mating can induce allopregnanolone synthesis; albeit, a question is the role of other reproductively-relevant behaviors, which may precede or follow mating, for allopregnanolone synthesis.

### Paced mating, more so than other reproductively-relevant behaviors, increases allopregnanolone synthesis

Reproductively-relevant behaviors are those that may improve reproductive success. For example, some of these reproductively-relevant behaviors are those that include increased exploration and reduced anxiety that would promote females leaving the natal nest and traversing a complex and novel environment to encounter other female conspecifics and potential mates for the first time. To address this in our laboratory, paced mating as well as measures of exploration (e.g., open field), anxiety (e.g., elevated plus maze), or social interaction with another female are assessed in a short battery of these tasks. Paced mating itself, or immediately following this battery of tasks, increases allopregnanolone synthesis in the midbrain, compared to testing in the battery without mating (Frye et al., 2007, 2014b). Together, these data support that mating can be utilized as a measure of allopregnanolone’s actions as well as a way to manipulate allopregnanolone levels in the midbrain. This model has then been used to assess the mechanisms of allopregnanolone, with a focus on non-traditional actions for mating and reproduction-relevant behaviors.

## Non-traditional actions of allopregnanolone in the midbrain VTA

Allopregnanolone has actions that are considered “non-traditional” when compared to actions peripherally secreted steroids have through binding to cognate steroid receptors in their distal target organs, including brain regions involved in reproductive and homeostatic processes, such as the hypothalamus, midbrain, and limbic system (Pfaff et al., 1976; Shughrue et al., 1997; Osterlund et al., 2000). These effects involve dimerization of the steroid bound receptor, DNA binding, mRNA transcription and translation, and, ultimately, protein expression that would alter the behavior of the cell/organism (often referred to as the “genomic” actions of steroids). It was believed that the shortest latency of when hormones are secreted and bind to receptors and initiate this intracellular process to ultimately alter behavior was on the order of tens of minutes (and even hours to days). This notion was challenged with the discovery of neurosteroidogenesis, by which steroids could be produced in the same tissue that they were having effects for behavior in, and that steroids could have such effects so rapidly that they cannot be explained by these genomic actions. To summarize decades of work by many laboratories, neuro(active)steroids, such as allopregnanolone, are known to have rapid effects, including those on neuronal excitability and synaptic function (Majewska et al., 1986; Morrow et al., 1987; Gee et al., 1995; Brot et al., 1997; Qiu and Lange, 2003; Weir et al., 2004; Lange, 2004; Belelli and Lambert, 2005; Skildum et al., 2005). These rapid effects are understood to involve direct or indirect modulation of ion-gated or other metabotropic neurotransmitter receptors, rather than traditional actions via cognate nuclear steroid hormone receptors; these actions are referred to as a novel or non-traditional actions of steroids. Indeed, many decades ago Hans Selye reported rapid anesthetic and anti-convulsive properties of allopregnanolone and other progestogens (Selye, 1941). In the decades following these observations, GABAergic mechanisms have been described for these anesthetic and anticonvulsant effects of allopregnanolone as well as some of the anxiolytic effects of allopregnanolone (Harrison and Simmonds, 1984; Majewska et al., 1986; Belelli and Lambert, 2005). For over two decades, our laboratory has been focused on GABA, dopamine, and glutamate as targets of allopregnanolone in the midbrain VTA for mating and reproductively-relevant responses.

### Allopregnanolone has actions via GABA, dopamine, and glutamate for mating

The VTA has rich innervation of dopamine targets and some of allopregnanolone’s actions in the VTA for mating may involve these targets, as well as GABA and glutamate. Progestogens can increase release of GABA, dopamine and glutamate (Lévesque and Di Paolo, 1990; Frye et al., 2000; Frye, 2001a,b). High levels of progestogens enhance number, density, and affinity of GABA_A_ receptors, coincident with enhancing lordosis (Mascó et al., 1986; Wilson, 1992; Frye and Vongher, 1999). There are D_1_ receptors on dopaminergic cell bodies and GABAergic terminals as well as NMDARs (Stoof and Kebabian, 1984; Bayer and Pickel, 1991; Willick and Kokkinidis, 1995). Greater GABA input onto GABA_A_ receptors that are located on GABAergic interneurons in the VTA mitigate inhibitory actions of these cells on dopamine cell bodies, thereby increasing dopamine release (from cell body and dendrites; Churchill et al., 1992). Excitation of D_1_ receptors on GABAergic afferents in the VTA increases GABA release (Kalivas and Duffy, 1995). Antagonists of D_1_ or GABA_A_ reduce allopregnanolone-facilitated lordosis when administered to the VTA and the opposite pattern is observed with agonists of D_1_ or GABA_A_ (Frye et al., 2004; Sumida et al., 2005; Frye and Paris, 2009). Furthermore, antagonists of GABA_A_ receptors to the VTA reduce allopregnanolone-facilitated lordosis, and the potentiation of this response by a D_1_ agonist co-administered to the VTA (Frye et al., 2006c). A role of N-methyl-D-aspartate receptor (NMDARs) is also suggested in this pathway. In support, D_1_ expressing GABAergic terminals, synapse on dopaminergic cell bodies that express both GABA_A_ receptors and NMDARs (Bayer and Pickel, 1991; Willick and Kokkinidis, 1995). Infusions of a NMDAR antagonist to the VTA increases allopregnanolone-facilitated reproductive responding of female rodents (Petralia et al., 2007; Frye and Paris, 2011). Together these findings suggest that allopregnanolone’s actions for reproductive responding in the VTA may be related to reductions in tonic inhibition of dopamine neurons in this region, involving actions of GABA_A_, D_1_, and NMDARs here. Additional studies have suggested downstream pathways for these receptors, including, including activity of G-proteins, adenylyl cyclase, phospholipase C and protein (a discussion of which is beyond the scope of this review, but can be found in Frye and Walf, 2008). Moreover, the functional role of membrane targets of the progestogens, such as the membrane progestin receptors, for reproductive indices have been shown across aquatic species and terrestrial mammals (Tokumoto, 2012; Tokumoto et al., 2012; Frye et al., 2013b, 2014c; Pang et al., 2013; Petersen et al., 2013; Schumacher et al., 2014); the extent to which there are interactions between these ionotropic and metabotropic targets in the VTA is of continued interest. A microarray analysis of gene expression changes in the midbrain of proestrous rats that had been paced mated or not confirmed the role of the targets involved in allopregnanolone metabolism and biosynthesis, as well as these neurotransmitter targets, but also revealed a novel target of interest, the pregnane xenobiotic receptor (PXR; Frye and Walf, 2008; Frye, 2009). The findings to date about this novel target in the midbrain are described as follows.

## Bridging sources and actions-role of PXR in the midbrain VTA

### PXR is expressed in the brain and may have hormone-relevant actions

A recent focus has been on characterizing the role of a promiscuous nuclear receptor, PXR, involved in cholesterol metabolism and expressed in the VTA, as a target for allopregnanolone and how this relates to both actions and production of allopregnanolone. PXR has well-known metabolic and clearance actions in the traditional organs for metabolism and excretion, such as the liver, kidneys, intestines, and the blood-brain barrier (Geick et al., 2001; Dussault and Forman, 2002; Kliewer et al., 2002; Francis et al., 2003; Bauer et al., 2004, 2006; Xu et al., 2005; Harmsen et al., 2007; Ma et al., 2008; Zhang et al., 2008; Ott et al., 2009). It is considered a promiscuous nuclear receptor with a long list of molecules that it positively modulates (including several steroids, and allopregnanolone) and much fewer molecules that are negatively modulated. Although early work on understanding the role of PXR outside of the liver and other excretory organs in the body was focused on the blood-brain-barrier, several laboratories, including our own, have demonstrated its expression in the brain proper (e.g., in rodents, rabbits, pigs, and humans; Bauer et al., 2004; Lamba et al., 2004; Marini et al., 2007; Mellon et al., 2008; Frye, 2011). In considering its role for metabolism and xenobiotic (including steroid) clearance in the liver, we sought to determine PXR’s functional effects related to allopregnanolone production and/or action. PXR protein and mRNA was expressed in the midbrain of proestrous rats, with higher expression (determined by western blots) in female rats in proestrous versus those in diestrous or male rats (Frye et al., 2012, 2013b), suggesting a possible role of ovarian steroids (estradiol, progesterone) and/or pregnane neurosteroids. These studies were correlational in nature and demonstrated a relationship between hormonal milieu and sex differences for expression of PXR.

### Manipulations of PXR in the midbrain for mating

Next, studies investigated manipulations of PXR in the midbrain for functional effects, including lordosis. Positive modulators of PXR, such as allopregnanolone, other pregnane steroids (3β,5α-THP, 3α,5β-THP), and RU486, when infused to the VTA, enhanced lordosis of OVX, estradiol-primed rats (Frye, 2011). However, these findings are tempered by the known promiscuity of PXR. Follow-up studies utilized a pharmacogenetic tool (antisense oligodeoxynucleotides, AS-ODNS) to reduce expression of PXR in the midbrain VTA to further understand functional outcomes (Frye, 2011; Frye et al., 2012, 2013a, 2014a,b). Investigations of the role of PXR, by using this PXR knockdown approach, for mating-induced neurosteroidogenesis and functional effects are ongoing, and some key findings are described as follows.

### Manipulations of PXR for allopregnanolone synthesis

**Figure 1:**
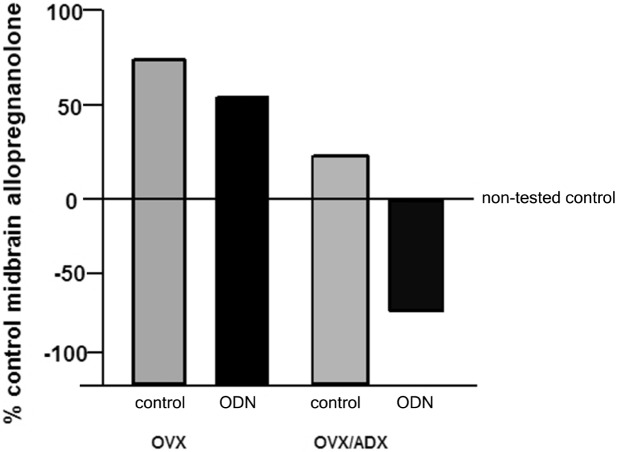
**Depicts the midbrain allopregnanolone levels of behaviorally tested animals compared to non-tested rats that were ovariectomized (OVX) or OVX and adrenalectomized (OVX/ADX), estradiol-primed and behaviorally tested in the paced mating task as a percent of the non-tested controls**. Rats in these conditions were also administered saline vehicle or pregnane xenobiotic receptor (PXR) antisense oligodeoxynucleotides (AS-ODNs) to the midbrain VTA. Paced mating increased midbrain allopregnanolone levels of rats compared to what was observed in the non-tested controls; this was attenuated with knockdown of PXR.

**Figure 2:**
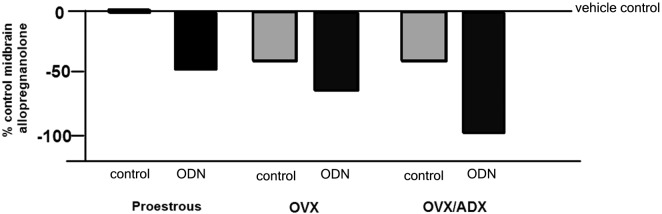
**Depicts the midbrain allopregnanolone levels of paced mated proestrous, ovariectomized (OVX) and estradiol-primed, or OVX and adrenalectomized (OVX/ADX) estradiol-primed rats infused with saline vehicle or pregnane xenobiotic receptor (PXR) antisense oligodeoxynucleotides (AS-ODNs) to the midbrain VTA**. Data are represented as a percent of the saline-infused proestrous control group. Administration of PXR AS-ODNs to the midbrain reduces allopregnanolone levels in the midbrain following mating across these hormone conditions.

The role of the PXR for biosynthesis of allopregnanolone in the brain has been investigated. An approach that was utilized to investigate this was to compare the capacity of rats with peripheral glands removed (e.g., the ovaries and/or the adrenal glands) to produce allopregnanolone in the midbrain following mating. In comparing rats that were OVX or OVX/ADX, estradiol-primed and behaviorally tested in the paced mating task to non-tested controls, there was a robust increase in midbrain levels of allopregnanolone, particularly among the OVX rats, with paced mating (Figure 1). This effect was attenuated when rats were administered PXR AS-ODNs to the midbrain VTA (Figure 1). Moreover, comparisons of rats that are paced mated and in different hormonal states (proestrous, OVX, OVX/ADX) and administered saline vehicle or PXR AS-ODN infusions to the midbrain VTA corroborate these findings. Administration of PXR AS-ODNs to the midbrain reduces allopregnanolone levels in the midbrain following mating across these hormone conditions (Figure 2). These data suggest a role of PXR for mating-induced allopregnanolone secretion in the midbrain VTA.

### PXR is upstream of TSPO for allopregnanolone synthesis

A question is how PXR may interact with other downstream factors recognized to be involved in neurosteroidogenesis. Investigation of this question has begun by assessing the role of TSPO, given that this is one rate-limiting factor for allopregnanolone synthesis in the brain. Inhibiting TSPO with PK11195 reduced allopregnanolone in the midbrain and lordosis, an effect that could be reversed with allopregnanolone replacement, but not when AS-ODNs and allopregnanolone were co-administered. AS-ODNs blocked actions of FGIN 1-27 for lordosis and allopregnanolone levels among proestrous > OVX > OVX/ADX rats. Together, these data support the notion that PXR may be upstream of TSPO. Investigations of the regulation of other related factors are underway.

### Interactions of PXR, glutamate and GABA receptors

**Figure 3:**
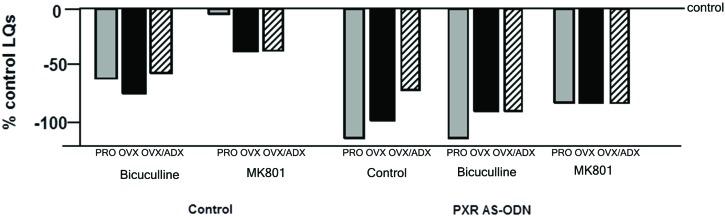
**Depicts the lordosis quotients of proestrous (PRO), ovariectomized (OVX) and estradiol-primed, or OVX and adrenalectomized (OVX/ADX) estradiol-primed rats infused with saline vehicle or pregnane xenobiotic receptor (PXR) antisense oligodeoxynucleotides (AS-ODNs) and then a GABA_A_ receptor antagonist (bicuculline), or a NMDAR antagonist (MK-801) to the midbrain VTA before behavioral testing**. Data are represented as a percent of the saline-infused proestrous control group. PXR AS-ODNs to the VTA potentiated these effects of GABA_A_ and NMDAR manipulations.

Although these data suggest that PXR is important for the synthesis of allopregnanolone in the midbrain, a related research question is the downstream factors for allopregnanolone’s actions. PXR AS-ODNs to the VTA, but not nearby midbrain sites, blocks reproductive responding among receptive rats associated with estrous cycle increases or following estradiol- and progestogen-administration to OVX rats (Frye et al., 2012, 2013a, 2014a). Moreover, knocking down expression of PXR in the midbrain VTA attenuates actions of allopregnanolone via NMDA and/or GABA_A_ receptors for lordosis (Figure 3). That there were some differences noted across hormonal milieu in this study, suggestive of a role of allopregnanolone biosynthesis, follow-up questions would include capacity for allopregnanolone biosynthesis in the brain as well as responses to allopregnanolone administration across these groups.

## Beyond synthesis and actions of allopregnanolone in the midbrain VTA

### Manipulations of PXR are most salient for socially-relevant behaviors

**Figure 4:**
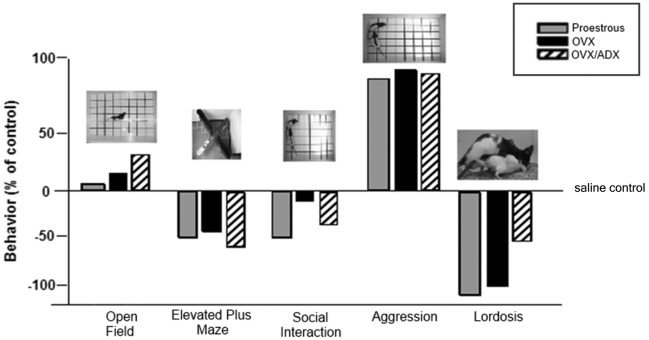
**Depicts the pattern of results for all behavioral measures in the testing battery (open field, elevated plus maze, social interaction and paced mating) of rats proestrous (PRO), ovariectomized (OVX) and estradiol-primed, or OVX and adrenalectomized (OVX/ADX) estradiol-primed rats infused with saline vehicle or pregnane xenobiotic receptor (PXR) antisense oligodeoxynucleotides (AS-ODNs) to the midbrain VTA**. Data are represented as a percent of the saline-infused control group for each respective hormone condition. Pictures of the tasks are included about the data bars. The most robust reduction in responding following PXR AS-ODNs infusions to the VTA were observed in the social interaction and paced mating task.

Another area of interest is the role of PXR for other socially-relevant behaviors. We have traditionally utilized studies such as those described above to ascertain mechanisms of allopregnanolone using lordosis in a mating task as one endpoint. Mating is typically assessed after other measures of behaviors that may support reproduction (i.e., reproductively-relevant behaviors, such as exploration, anxiety, and pro-social behavior) that allopregnanolone mediates (Frye, 2011; Frye et al., 2012, 2013a,b), perhaps through its actions at GABA_A_ receptors and NMDARs (Frye and Paris, 2009, 2011). In comparing the extent to which PXR AS-ODNs reduce such behaviors, we have consistently noted that the most robust effects are for lordosis quotients, followed by other socially-relevant measures (aggression/rejection during the mating task, and social investigation of a female conspecific), and then affective measures (open arm exploration in the plus maze) and then exploratory/ambulatory behavior (open field entries made; Figure 4). These data suggest overall that manipulations of PXR are most salient for socially-relevant behaviors, and that the midbrain infusions of such drug manipulations are not associated with non-specific effects of ambulatory behavior.

### Brain targets beyond the midbrain VTA

These comparisons suggest the specificity of the response as well as brain targets outside of the midbrain VTA. Among receptive rats, mating-induced allopregnanolone synthesis is observed in the midbrain as well as corticolimbic structures (hippocampus, prefrontal cortex) and the hypothalamus (Frye et al., 2006a, 2007). PXR AS-ODNs to the midbrain VTA of receptive rats have the most salient effects to reduce allopregnanolone in the midbrain, but reductions are also observed in the hippocampus (Frye et al., 2013a, 2014a). Interestingly, PXR AS-ODNs to the midbrain VTA also reduce levels of the growth factor, brain-derived neurotrophic factor (BDNF), in the hippocampus coincident with differences in behavior (Frye et al., 2014b). Allopregnanolone has actions on BDNF as well as cognitive performance of rodents (Nin et al., 2011; Frye et al., 2013a; Bali and Jaggi, 2014). As such, the extent to which PXR is a target of allopregnanolone beyond the midbrain to corticolimbic structures is of great interest. Indeed, genetic knockout of a related nuclear receptor known for its actions in the liver, the liver X receptor, increases anxiety-like behavior of mice and alters GABAergic function in the hypothalamus, as well as may play a protective role in a Parkinson’s disease mouse model (Dai et al., 2012; Tan et al., 2012). Thus, we consider that allopregnanolone may have a role via PXR in the midbrain and beyond for neural and behavioral plasticity.

### Mating behavior of PXR knockout rats and mice

**Figure 5:**
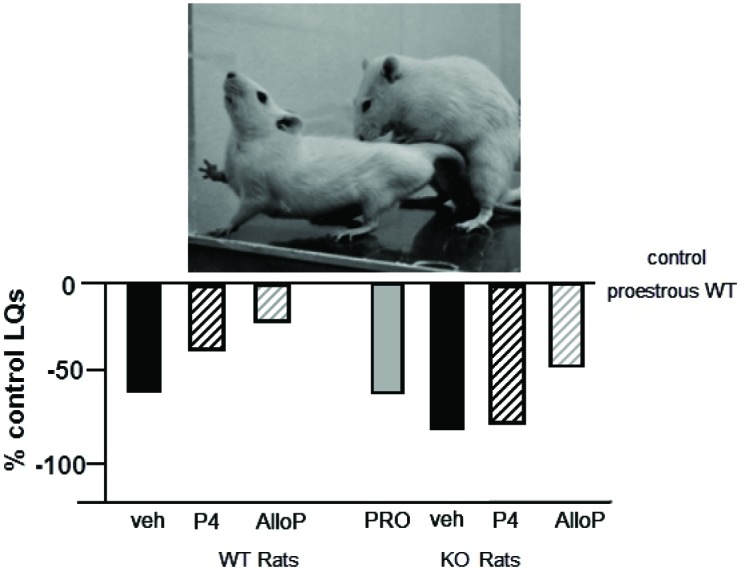
**Depicts the lordosis quotients of proestrous (PRO; positive control) and ovariectomized (OVX) rats administered vehicle, progesterone (P_4_) and/or allopregnanolone (AlloP) via subcutaneous injections**. Comparisons were made in Sprague-Dawley wildtype (WT) and pregnane xenobiotic receptor (PXR) knockout (KO) rats on a Sprague-Dawley background. This strain of rats is commercially available from SAGE Labs. Data are represented as a percent of the WT proestrous control group. A picture of lordosis is included above the data bars. PXRKO rats have lower lordosis than do WT rats across hormone conditions, with similar improvements in lordosis with replacement back of AlloP.

**Figure 6:**
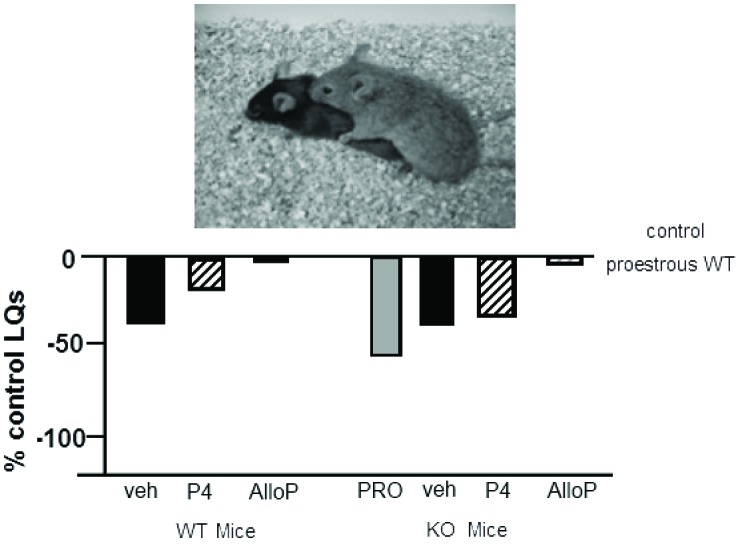
**Depicts the lordosis quotients of proestrous (PRO; positive control) and ovariectomized (OVX) mice administered vehicle, progesterone (P_4_) and/or allopregnanolone (AlloP) via subcutaneous injections**. Comparisons were made to C57BL/6Tac wildtype (WT) and pregnane xenobiotic receptor (PXR) knockout (KO) mice on a C57BL/6Tac background. This strain of mice is commercially available from Taconic. Data are represented as a percent of the WT proestrous control group. A picture of lordosis is included above the data bars. PXRKO mice have lower lordosis than do WT mice across hormone conditions, except for with replacement back of AlloP.

We have begun characterizing the role of lifelong knock down of PXR as well as species similarities/differences, using PXR knockout (KO) rats and mice. Progesterone administration produced similar rates of lordosis as observed among proestrous wildtype (WT), but not PXRKO, rats; both WT and PXRKO rats responded to allopregnanolone administration with increased lordosis (Figure 5). The same pattern was observed among WT and PXRKO mice, suggesting species similarities in this mechanism (Figure 6). These data corroborate what has been observed with PXR knockdown in the VTA of rats to reduce lordosis of receptive rats, but show that PXRKO rats can respond to allopregnanolone administration (unlike what has been observed with allopregnanolone infusions to the VTA following PXR AS-ODN infusions; Frye et al., 2014a). These results are promising in that they suggest a specific deficit in synthesis of allopregnanolone, rather than binding of allopregnanolone as just one of many, many positive modulators of this promiscuous nuclear receptor. However, it is not known what the capacity for mating-induced allopregnanolone, and whether there are similar brain targets, is in these rodent models at this time. As well, a typical concern with models of whole body and brain knockout of a gene throughout development is the potential for compensatory mechanisms. Studies are ongoing to characterize these animal model resources further.

### Other behavioral phenotypes in the PXR knockout rats and mice—the mirror maze

Of interest is whether there are other behavioral phenotypes in the PXRKO rats and mice to consider. There are no apparent differences in their homecage behavior, and systematic analyses are underway to assess other behavioral endpoints. A prediction, based upon the data with PXR AS-ODNs, is that the most salient effects of PXR knockout may be for reproductive measures (as supported by data in Figures 5 and 6) or social behaviors. Indeed, a pilot assessment of WT and PXRKO mice in the mirror maze supports this notion. The mirror maze is a behavioral assessment of acute changes in rodents’ responses to observations of their own image in a mirror (Houri, 1986; Lamberty, 1998). This task, like several others considered to be an index of affective responsing (e.g., elevated plus maze), is considered a free-choice conflict task in which the time spent by the rodent in the mirrored section of a cubed chamber is compared to the time spent away from the mirrors in an adjoined alleyway without mirrors (Henderson et al., 2004; Frye et al., 2006b). We have utilized this task to assess the role of allopregnanolone and other steroid targets, including the androstane equivalent of allopregnanolone (3α-androstanediol; Frye et al., 2006b, 2008; Walf et al., 2009). In initial assessments of female WT and PXRKO mice during the proestrus phase of the estrous cycle, we noted an approximately 15% increase in time spent in the mirror chamber among the PXRKO mice (197 s) than in the WT mice (172 s). This pattern is opposite to what has been noted with PXR knockdown or knockout for interaction with a mate or conspecific (as described above). As well, female mice with knockout of estrogen receptor beta, which may be another important factor in allopregnanolone synthesis, but not progestin receptor, respond poorly in this task. These data in the mirror maze are interesting as they suggest a role of PXR for mediating responses to another socially-relevant stimuli, the rodents’ own image in a mirror, beyond a mate (as in the paced mating task) or another female conspecific (as in the social interaction task). Although a focus has been on actions of allopregnanolone in the midbrain VTA, how the understanding of these novel targets in this region can be extended elsewhere in the CNS relevant for clinical conditions is of continued interest.

## Beyond homeostasis—allopregnanolone’s role in translation

Diverse functions have been ascribed to the actions of allopregnanolone, including many of the actions described above for reproduction and other reproductively-relevant behaviors. Our understanding of allopregnanolone’s actions in the VTA for lordosis has been extended to reveal the role of allopregnanolone for broader, clinically-relevant questions, such as neurodevelopmental processes, neuropsychiatric disorders, epilepsy, and aging (reviewed in Frye, 2009). Some examples about the role of allopregnanolone for seizure and affective processes in clinical populations are as follows. Large clinical trials and a case study support that allopregnanolone may be involved in seizure control (Herzog and Frye, 2003; Herzog et al., 2006, 2014). There are mediating effects of allopregnanolone for anxiety and depressive symptoms among women with premenstrual dysphoric disorder (Endicott et al., 1999; Freeman et al., 2002; Gracia et al., 2009) as well as self-reported anxiety in men with post-traumatic stress disorder following exposure to trauma cue (Casada et al., 1998; Frye, 2009). Furthermore, allopregnanolone may underlie some of the effects of therapeutics. Fluoxetine can enhance dihydroprogesterone (DHP)’s affinity for 3α-HSD, thereby increasing allopregnanolone formation (Griffin and Mellon, 1999). Reductions in depressive symptoms of men or women diagnosed with major depression are correlated with higher cerebrospinal fluid levels of allopregnanolone (Romeo et al., 1998; Uzunova et al., 1998). Thus, biosynthesis and subsequent rapid effects of allopregnanolone at its non-traditional targets (GABA_A_, glutamate, dopamine, and PXR) are mechanisms of continued interest with respect to these clinical conditions.

## Summary and conclusions

**Figure 7:**
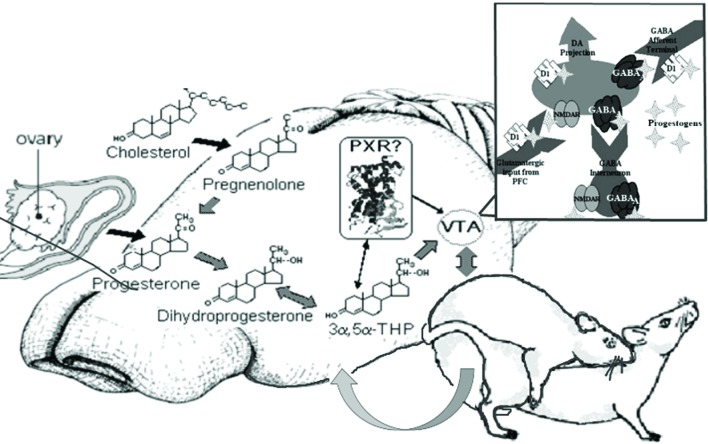
**Model of proposed actions of allopregnanolone produced in the brain via PXR and other novel targets, important for reproductive responding**. In summary, our lab has been focused on elucidating the novel targets of allopregnanolone, such as PXR, for its functional effects. The data to date suggest that PXR is a novel target of allopregnanolone in the midbrain VTA, as are neurotransmitter targets (e.g., GABA, NMDA), for functional, reproductively-relevant responses, as well as biosynthesis of allopregnanolone in the brain (i.e., acting upstream of TSPO).

In summary, investigations of allopregnanolone’s production and function in the midbrain VTA have focused on mating as a measure and manipulation of allopregnanolone. First, there are traditional (metabolism from peripheral steroids) and nontraditional (biosynthesis, or production in the brain from cholesterol, following challenges such as mating) means for production of allopregnanolone in the central nervous system. Second, the non-traditional mechanisms in the brain that allopregnanolone has for behavioral processes, including mating and reproduction-relevant behaviors, depends upon rapid modulation of neurotransmitters (GABA, glutamate, dopamine), instead of binding to steroid receptors. Third, PXR is a target bridging the synthesis of allopregnanolone with its functions in brain and may be upstream of TSPO and modulate actions of allopregnanolone via neurotransmitter targets (Figure 7). Fourth, the significance of studying the functions and mechanism of allopregnanolone in VTA can be extended to clinically-relevant findings for neuropsychiatric, neurodevelopmental, neurodegenerative, and/or age-related disorders. In conclusion, neurosteroids have novel actions, which are now well-accepted, related to their production in the brain and their actions through non-steroid receptor targets. Future considerations include further understanding another characteristic of neurosteroids, which their capacity to induce steroidogenic enzymes in the brain, and thus be involved in clearance (as is PXR). As such, the role of PXR as a factor involved in steroid production, action, *and* clearance in the brain is of continued study in our laboratory.

## Author contributions

All authors on this paper substantially contributed to the work reviewed herein and the composition of this manuscript. Carolyn J. Koonce was involved in acquisition, analysis, and interpretation of data represented in figures, and drafting of figures for paper, the reference list and editing this entire work. Alicia A.Walf was involved in acquisition, analysis, and interpretation of data represented in figures, and drafting, editing, and revising of all sections of the paper. Cheryl A. Frye was involved in the conception and study design, acquisition, analysis, and interpretation of data of all studies in the lab described, reviewing, editing, and drafting versions of the work, and giving final approval of the paper to be submitted.

## Conflict of interest statement

The authors declare that the research was conducted in the absence of any commercial or financial relationships that could be construed as a potential conflict of interest.
